# Thunder and Lightning

**Published:** 1884-06

**Authors:** 


### THUNDER AND LIGHTING.

WE visit the mountains and take in the inspiring view, with no fear of harm, though the avalanche may have swept their ridges in years past; we stand safely on the shore and study with awe the huge waves that continuously dash at our feet, or we witness the unutterable grandeur of the North, with no mortal fear to mar our peace or divert our meditation, and yet some of us may sit in our homes and quake when one of the grandest scenes of nature comes to our view.

It need not be so; the laws that govern a thunder storm—the passage of the electric current between clouds and the earth—are as fixed, and as capable of being understood and taken advantage of, as any of the natural laws governing other phenomena in our world.

Lightning will not play about a house in a fickle frolic, and then jump out the window, as some newspaper reports would suggest, nor will it roll about as a ball of fire, explode and curiously disappear, as some imagine. This supposed sight is an optical delusion, as truly as that of the sun rising and setting. Lightning is too quick to be seen in its passage.

We need never fear the flash we *see;* for when seen the danger has passed!

So much for the negative effects—now for a few positive, which, if heeded, may result practically in protecting ourselves and homes.

If a water pipe bursts in your residence, you look for the escape of water, and can anticipate its course from room to room till it reaches the lowest level; so the course of an electric discharge can be previously determined; not by the law of gravitation, but by as sure laws of conductivity and resistance.

The lightning, in striking any building, tree, or other high object, is simply seeking a passage to the moist earth; and it will find and take the best conductor near its course, following a poor conductor only when a better one is wanting, and only till a better one can be reached. All metals are good conductors, living animals are tolerably good, much better than dry wood or air; moist wood, the soot in chimneys or any moist substance, are better than air.

Buildings that are piped throughout for water or gas are already well protected against danger to people within; for if the pipes extend above the rooms, no lightning that may strike the house will seriously affect the persons within. It will find the pipes if any are in the locality of the discharge and pass through them silently to the earth. For this reason, but few people in city houses are killed by lightning, though the top of the building may be shattered.

A lightning rod, as ordinarily constructed, may be a partial protection, but owing to the fact that they generally are stuck only a few feet, or sometimes inches in the comparatively dry earth, they are not to be depended upon.

The most simple and reliable method of protecting a building that is piped for water or gas, is to connect all metal about the roof such as tin roof, metal water conductors, fancy iron work, &c., by means of a one quarter inch copper wire, soldered to the roof metals and to the nearest water or gas pipe. It is well also, to have a piece of copper wire extend up any chimney or high point on the roof, the lower end connecting with the tin roof or the main wire leading to the pipe. These wires can safely pass down through the building, for, as before stated, lightning will not leave such a conductor for a poorer one. Glass insulators on lightning rods are of no use; if the rods are in proper condition the discharge of electricity will not leave them for the house, and if the rods are not all right it will leave them at a point nearest a better conductor, no matter how much glass may intervene.

If the gas, instead of water pipe, is to be used as the lower terminal of the protecting rod, the connection should be made at a point between the meter and street, as the meter may at some time be removed, and thus sever the metallic connection.

In a building not supplied with pipes, the large copper wire should extend to all high points, and connect with all large quantities of metal about the roof, as in the former case, with its lower end attached to a quantity of old metal of any kind, equal to twenty-five or fifty square feet of surface, well buried in the moist earth. This wire can also safely pass down through the building, or be "stapled" to any convenient part of the house. A large building should have more than one wire passing to the earth.

Iron rods can be used, but they must be about four times the size of copper to be equally as good, and are harder to bend and solder.

Do not suppose that because water pipes are named, your connection may be made with a pipe passing only to a cistern in the house. A considerable extent of metal surface in permanently moist earth is the only proper connection for the rod or conductor.

A metallic connection between the tin roof of a railroad car and the axle box, would prevent injury to passengers by lightning.

Telephone and telegraph wires are a protection rather than otherwise, for their ends connect with the earth. A building over which a sufficient number of such wires pass to conduct a large quantity of electricity, would be protected.

We should not try to insulate ourselves by putting glass knobs on our chairs, or sleeping on feather beds! But take the opposite course and give the electric current a substantial, ample and easy passage to the deep, moist earth; then *enjoy* a thunder storm as we would other sublime works of the Creator.—*Portland Transcript.*

